# Draft Genome Sequences of 40 Dermatophilus congolensis Isolates from Bovine Dermatophilosis Cases in St. Kitts and Nevis

**DOI:** 10.1128/MRA.00334-21

**Published:** 2021-05-27

**Authors:** Ian Branford, Shevaun Johnson, Samantha Zayas, Aspinas Chapwanya, Filip Boyen, Matylda B. Mielcarska, Lidia Szulc-Dąbrowska, Patrick Butaye, Felix N. Toka

**Affiliations:** a Ross University School of Veterinary Medicine, Basseterre, St. Kitts and Nevis; b Ghent University, Faculty of Veterinary Medicine, Department of Pathology, Bacteriology, and Avian Diseases, Merelbeke, Belgium; c Warsaw University of Life Sciences-SGGW, Institute of Veterinary Medicine, Warsaw, Poland; University of Maryland School of Medicine

## Abstract

Dermatophilus congolensis causes dermatophilosis in cattle, mainly in tropical climates. Despite the economic losses caused by this bacterium, its pathogenic factors are less well understood. We report draft genomes of D. congolensis strains isolated during a dermatophilosis outbreak in cattle in St. Kitts and Nevis. Some isolates contain *tet*(Z), which is responsible for resistance to tetracyclines.

## ANNOUNCEMENT

Dermatophilus congolensis belongs to the family *Dermatophilaceae* in the genus *Dermatophilus*. It impacts animal health, reduces animal productivity, results in carcass condemnation, and damages the hide. There is no vaccine against this zoonotic pathogen, which may be underdiagnosed in humans (1). The rationale for sequencing is that limited data exist on the molecular characteristics of the D. congolensis genome and the 2019 outbreak of disease included animal deaths, suggesting a role for potential emerging virulent strains. We collected 78 scab samples from cattle during a dermatophilosis outbreak in St. Kitts. To isolate the bacteria, the scabs were placed in a sterile mortar and crushed with a pestle. Fragments were transferred to 2 ml of sterile distilled water in a tube and left at room temperature for 3 h. Later, the tube was placed unsealed in a jar with a lit candle for 15 min. A loopful of solution from the surface of the tube contents was inoculated onto blood agar plates, incubated for up to 72 h at 37°C in 5% CO_2_, and inspected for growth daily. Suspected colonies, i.e., small, yellow, raised, β-hemolytic, and embedded in agar after 24 to 48 h, were subcultured on blood agar until a pure culture was obtained. To obtain material for identification and genomic DNA isolation, bacteria were cultured in liquid brain heart infusion broth for 48 h at 37°C in 5% CO_2_. Bacterial isolates were identified as D. congolensis by matrix-assisted laser desorption ionization–time of flight mass spectrometry (MALDI-TOF MS) (2). Additionally, PCR confirmed the presence of the *agac* gene, which encodes the D. congolensis alkaline ceramide protein (3).

Bacterial DNA was prepared with the AllPrep bacterial DNA/RNA/protein kit (Qiagen, USA). The quality and concentration of DNA were determined with a Qubit 2.0 fluorometer with the following optical density (OD) ratios: OD at 260 nm (OD_260_)/OD_280_ of 1.9 and OD_260_/OD_230_ of 1.9. Libraries were prepared using the Nextera XT library preparation kit and were sequenced on an Illumina HiSeq system using the 2 × 150-bp strategy. Libraries were sequenced at Admera Health, LLC (South Plainfield, NJ, USA). The genomes were sequenced at a coverage of ≥30×. Quality control of derived sequences was performed with FastQC v0.11.5 (4) (https://narrative.kbase.us). Adapters and low-quality reads were removed with TrimGalore v0.6.4 (https://github.com/FelixKrueger/TrimGalore) with the following parameters: retain reads with a Phred quality score above 30, discard reads whose length is less than 50 bp after quality control, and maintain paired-end read order. Genome assembly was performed with SPAdes v3.13.0 (https://narrative.kbase.us) or MEGAHIT v1.1.1, and the assembly quality was determined with QUAST v4.4 (5). Annotation was performed with the NCBI Prokaryotic Genome Annotation Pipeline (PGAP) v4.11 (6). GenBank and SRA accession numbers for each isolate and assembly statistics are presented in Table 1. For easy identification, we have named the isolates BTSK followed by a number.

**TABLE 1:** Statistics for draft genome assemblies of D. congolensis strains isolated from acute cases of dermatophilosis on the island of St. Kitts

Isolate name	Species	GenBank accession no.	SRA accession no.	Total no. of reads	Final coverage (×)	*N*_50_ (bp)	Genome size (bp)	No. of contigs	No. of coding sequences	G+C content (%)
BTSK1	D. congolensis	JAAFOV000000000	SRX7208580	1,977,952	225	62,641	2,636,036	2	2,482	58.8
BTSK2	D. congolensis	JAAFOU000000000	SRX7208581	2,047,090	233	36,669	2,635,845	2	2,501	58.8
BTSK3	D. congolensis	JAAFOT000000000	SRX7208592	2,181,622	247	36,669	2,645,506	4	2,506	59.2
BTSK4	D. congolensis	JAAFOS000000000	SRX7208603	2,005,910	228	36,032	2,644,574	6	2,504	59.2
BTSK5	D. congolensis	JAAFOR000000000	SRX7208614	2,376,664	263	35,808	2,707,787	4	2,566	59.2
BTSK6	D. congolensis	JAAFOQ000000000	SRX7208615	2,071,660	230	36,699	2,707,101	4	2,580	59.1
BTSK7	D. congolensis	JAAFOP000000000	SRX7208616	2,023,060	230	46,334	2,634,952	2	2,546	58.8
BTSK8	D. congolensis	JAAFOO000000000	SRX7208617	2,081,650	239	39,504	2,610,909	2	2,530	58.8
BTSK9	D. congolensis	JAAFON000000000	SRX7208618	1,964,496	218	34,393	2,705,920	5	2,577	59.1
BTSK10	D. congolensis	JAAFOM000000000	SRX7208619	2,086,030	231	36,071	2,706,861	7	2,581	59.1
BTSK11	D. congolensis	JAAFOL000000000	SRX7208582	1,977,630	219	34,352	2,704,542	9	2,564	59.1
BTSK12	D. congolensis	JAAFOK000000000	SRX7208583	1,940,046	215	35,040	2,706,162	8	2,567	59.1
BTSK13	D. congolensis	JAAFOJ000000000	SRX7208584	2,298,664	255	39,414	2,705,629	6	2,562	59.1
BTSK14	D. congolensis	JAAFOI000000000	SRX7208585	1,947,074	216	36,055	2,706,605	5	2,520	59.1
BTSK15	D. congolensis	JAAFOH000000000	SRX7208586	1,914,556	212	35,867	2,706,691	4	2,553	59.1
BTSK16	D. congolensis	JAAFOG000000000	SRX7208587	2,085,600	231	35,425	2,704,123	4	2,561	59.1
BTSK17	D. congolensis	JAAFOF000000000	SRX7208588	1,974,306	225	40,401	2,635,364	2	2,551	58.8
BTSK18	D. congolensis	JAAFOE000000000	SRX7208589	2,022,336	230	34,301	2,643,372	3	2,486	59.2
BTSK19	D. congolensis	JAAFOD000000000	SRX7208590	2,102,574	238	36,056	2,646,426	5	2,505	59.2
BTSK20	D. congolensis	JAAFOC000000000	SRX7208591	1,835,522	208	40,084	2,643,778	3	2,498	59.2
BTSK21	D. congolensis	JAAFOB000000000	SRX7208593	2,478,552	285	43,043	2,609,406	3	2,513	58.8
BTSK22	D. congolensis	JAAFOA000000000	SRX7208594	2,125,938	236	35,872	2,705,748	4	2,584	59.1
BTSK23	D. congolensis	JAAFNZ000000000	SRX7208595	1,812,266	201	36,023	2,707,702	5	2,584	59.1
BTSK24	D. congolensis	JAAFNY000000000	SRX7208596	2,157,370	245	36,132	2,644,395	4	2,480	59.2
BTSK25	D. congolensis	JAAFNX000000000	SRX7208597	1,876,270	213	39,840	2,644,659	4	2,481	59.2
BTSK26	D. congolensis	JAAFNW000000000	SRX7208598	2,120,640	236	40,081	2,690,948	5	2,546	59.1
BTSK27	D. congolensis	JAAFNV000000000	SRX7208599	1,990,734	227	42,739	2,635,575	2	2,505	58.8
BTSK28	D. congolensis	JAAFNU000000000	SRX7208600	2,055,712	233	36,892	2,644,675	4	2,536	59.2
BTSK29	D. congolensis	JAAFNT000000000	SRX7208601	2,009,138	228	35,918	2,641,543	5	2,518	59.2
BTSK30	D. congolensis	JAAFNS000000000	SRX7208602	2,059,488	229	37,662	2,692,568	4	2,526	59.1
BTSK31	D. congolensis	JAAFNR000000000	SRX7208604	1,800,378	205	38,314	2,635,532	2	2,536	58.8
BTSK32	D. congolensis	JAAFNQ000000000	SRX7208605	2,589,746	294	52,013	2,640,714	3	2,499	59.2
BTSK33	D. congolensis	JAAFNP000000000	SRX7208606	1,724,744	196	35,444	2,640,994	4	2,490	59.2
BTSK34	D. congolensis	JAAFNO000000000	SRX7208607	1,967,536	224	44,591	2,635,305	2	2,527	58.8
BTSK35	D. congolensis	JAAFNN000000000	SRX7208608	1,965,800	223	36,107	2,642,663	2	2,488	59.2
BTSK36	D. congolensis	JAAFNM000000000	SRX7208609	1,928,268	218	35,350	2,649,493	4	2,496	59.2
BTSK37	D. congolensis	JAAFNL000000000	SRX7208610	1,958,350	222	36,093	2,642,605	4	2,517	59.2
BTSK38	D. congolensis	JAAFNK000000000	SRX7208611	1,940,290	220	38,501	2,640,313	3	2,503	59.2
BTSK39	D. congolensis	JAAFNJ000000000	SRX7208612	1,781,368	202	34,368	2,640,884	4	2,540	59.2
BTSK40	D. congolensis	JAAFNI000000000	SRX7208613	1,999,598	227	38,550	2,640,858	4	2,510	59.2

We used the Comprehensive Antibiotic Resistance Database (CARD) v3.0.7 (Resistance Gene Identifier [RGI] v5.1.0; https://card.mcmaster.ca/analyze/rgi) (7) and ResFinder v3.2 (https://cge.cbs.dtu.dk/services/ResFinder) (8) to search for antimicrobial resistance genes. The two databases identified the *tet*(Z) gene in 12 isolates of D. congolensis. The gene confers resistance to tetracyclines. PHASTER (BLAST+ v2.3.0+) (https://phaster.ca) (9) identified prophage sequences.

### Data availability.

The whole-genome sequencing data and raw read data have been deposited in GenBank under the BioProject accession number PRJNA590056. Table 1 lists the accession numbers for draft genome assemblies and Sequence Read Archive (SRA) data.
